# Endogenous cell wall degrading enzyme LytD is important for the biocontrol activity of *Bacillus subtilis*

**DOI:** 10.3389/fpls.2024.1381018

**Published:** 2024-04-10

**Authors:** Luotao Wang, Jianquan Huang, Si Chen, Xin Su, Xun Zhang, Lujun Wang, Wei Zhang, Zhenshuo Wang, Qingchao Zeng, Qi Wang, Yan Li

**Affiliations:** ^1^ Department of Plant Pathology, College of Plant Protection, China Agricultural University, Beijing, China; ^2^ The Research Institute of Forestry and Pomology, Tianjin Academy of Agricultural Sciences, Tianjin, China; ^3^ Airport Research Institute, China Academy of Civil Aviation Science and Technology, Beijing, China; ^4^ Weinan Grapevine Research Institute, Weinan, China

**Keywords:** autolysin, peptidoglycan, cell wall degrading enzyme, colonization, biofilm, induced resistance, biological control

## Abstract

Autolysins are endogenous cell wall degrading enzymes (CWDEs) in bacteria that remodel the peptidoglycan layer of its own cell wall. In the *Bacillus subtilis* genome, at least 35 autolysin genes have been identified. However, the study of their roles in bacterial physiology has been hampered by their complexity and functional redundancy. *B. subtilis* GLB191 is an effective biocontrol strain against grape downy mildew disease, the biocontrol effect of which results from both direct effect against the pathogen and stimulation of the plant defense. In this study, we show that the autolysin *N*-acetylglucosaminidase LytD, a major autolysin of vegetative growth in *B. subtilis*, plays an important role in its biocontrol activity against grape downy mildew. Disruption of *lytD* resulted in reduced suppression of the pathogen *Plasmopara viticola* and stimulation of the plant defense. LytD is also shown to affect the biofilm formation and colonization of *B. subtilis* on grape leaves. This is the first report that demonstrates the role of an endogenous CWDE in suppressing plant disease infection of a biological control microorganism. These findings not only expand our knowledge on the biological function of autolysins but also provide a new target to promote the biocontrol activity of *B. subtilis*.

## Introduction

As the demand increases for more environmentally friendly disease control alternatives to the massive use of chemical pesticides, the interest in identifying and developing effective biological control agents of plant diseases has significantly increased in the last decades (Koch et al., 2018; Collinge et al., 2022; Lahlali et al., 2022). Bacteria from genus *Bacillus* are considered as one of the most important microorganisms in biological control against plant diseases of various crops (Fira et al., 2018; Villarreal-Delgado et al., 2018; Etesami et al., 2023). Besides the high efficacy against phytopathogens, additional advantages of using these bacteria include the ease of culture, storage, and manufacture due to the ability of *Bacillus* to produce endospores (Biermann and Beutel, 2023). In recent years, commercial formulated *Bacillus*-based biological pesticides have been increasingly used to control various plant diseases worldwide. To date, *B. subtilis* is among the most exploited species from the genus (Miljaković et al., 2020).

*B. subtilis* has been reported to be effective in controlling plant diseases through diverse mechanisms, including both direct and indirect mechanisms. Numerous studies revealed that *B. subtilis* protects plants against pathogens via a direct antagonistic effect on the pathogens by producing diverse antimicrobial compounds. The three families of *Bacillus* cyclic lipopeptides (CLPs)—surfactins, iturins, and fengycins—were mostly studied as bioactive compounds due to their antagonistic activity for a wide range of potential phytopathogens, including bacteria, fungi, and oomycetes (Ongena and Jacques, 2008). The antimicrobial activity of *B. subtilis* could also be due to the production of cell wall degrading enzymes (CWDEs) such as chitinases, glucanases, and proteases, which efficiently hydrolyze the major components of the fungal and bacterial cell walls and limit their growth or activity (Miljaković et al., 2020). The biocontrol activity exhibited by *B. subtilis* can also be attributed to indirect mechanisms, including biofilm formation, plant growth promotion (PGP), competition for nutrients and colonization sites, and induced systemic resistance (ISR) (Hashem et al., 2019). Besides antimicrobial activity, *Bacillus* CLPs also influence the colonization and have a key role in the beneficial interaction of *B. subtilis* with plants by stimulating host defense mechanisms (Ongena and Jacques, 2008; Fan et al., 2017). *B. subtilis* forms biofilms on the plant, which help to produce lipopeptides and augment their activity (Zeriouh et al., 2014). In addition, *B. subtilis* could also secrete CWDEs, such as cellulases, hemicellulases, and pectinases, to degrade plant cell wall. The release of cell-wall-derived oligosaccharides with danger-associated molecular pattern (DAMP) capacity could trigger a signaling cascade that leads to the induction of defense responses (Héloir et al., 2019; Miljaković et al., 2020).

One prerequisite of effective biocontrol is the successful colonization of the biocontrol microorganism on plants. Bacteria associated with plant leaves employ a variety of colonization strategies. Steps in these colonization strategies include modification of their environment on leaf, aggregation, ingression, and egression. The formation of bacterial aggregates may enhance the local habitat modification on the surface of leaves (Beattie and Lindow, 1999). In nature, approximately 70% of bacteria live in the form of aggregates on leaves, which confers them a selective advantage for survival and colonization (Monier and Lindow, 2003). Biofilms, which are communities of aggregated cells embedded in a self-produced extracellular polymeric matrix, are critical for *Bacillus* spp. colonization efficiency and thus the suppression of pathogens (Pandin et al., 2017; Fessia et al., 2022).

The cell wall, which can be found in archaea, bacteria, fungi, plants, and algae, is a complex and selectively permeable layer that surrounds the cell with main functions to provide protection structure and support to the cell (Keegstra, 2010; Free, 2013; Giovannoni et al., 2020). Therefore, the cell wall is resistant to various biotic and abiotic stresses. In this respect, “attacking” organisms evolved various enzymes specialized in cell wall degradation, which are known as exogenous CWDEs. Additionally, organisms contain endogenous CWDEs to remodel their own cell wall structures during development (Giovannoni et al., 2020). These degrading enzymes in bacteria are also collectively referred to as autolysins, which are found in all bacteria that digest the shape-maintaining and stress-bearing peptidoglycan layer of its own cell wall (Smith et al., 2000; Nayyab et al., 2017). Despite extensive researches on CWDEs, much of them focused on exogenous CWDEs, which evolved from organisms to degrade the cell wall of their targets. Few studies, especially in bacteria, have investigated the physiological functions of endogenous CWDEs that remodel their own cell wall structures during development.

Peptidoglycan, the major structure component of bacterial cell wall and the substrate of autolysins, is an alternating heteropolymer of *N*-acetylglucosamine (GlcNAc) and *N*-acetylmuramic acid (MurNAc) with pentapeptide side chains branching from the MurNAc residues. It is a dynamic structure continually being synthesized, modified, and hydrolyzed to allow for cell growth and division (Angeles and Scheffers, 2021). In the *B. subtilis* genome, at least 35 autolysin genes have been identified. The autolysins can be divided into four classes based on their cleave sites: *N*-acetylglucosaminidases, muramidases, acetyl-muramyl-L-alanine amidases (amidases), and endopeptidases (Ghuysen et al., 1966; Smith et al., 2000; Nayyab et al., 2017; Angeles and Scheffers, 2021). Among them, *N*-acetylglucosaminidase LytD (or CwlG) and amidase LytC (or CwlB) are two major autolysins of vegetative growth in *B. subtilis*, and these two autolysins bear 95% of the autolytic activity of the cells (Smith et al., 2000; Horsburgh et al., 2003; Ren et al., 2022). The autolysins associated with *B. subtilis* were shown to play a role in several important cellular functions, including differentiation, cell lysis, cell wall growth and turnover, cell separation, competence, and motility (Smith et al., 2000). However, the study of their physiological roles has been hampered by their complexity and functional redundancy.

LytD is a key *N*-acetylglucosaminidase acting on peptidoglycan in *B. subtilis* (Smith et al., 2000; Horsburgh et al., 2003; Nayyab et al., 2017). Previous reports showed that inactivation of *lytD* alone did not affect cell separation, autolysis, cell wall turnover, growth rate, competence, and sporulation (Margot et al., 1994). It is required for the motility function but only in concert with LytC (Rashid et al., 1995). Despite the fact that LytD has been identified for many years, its biological function remains unclear. *B. subtilis* GLB191 is an efficient biocontrol strain against grape downy mildew disease caused by the pathogen *Plasmopara viticola* (Zhang et al., 2017). Our previous report showed that the biocontrol effect of *B. subtilis* GLB191 on grape downy mildew disease is through direct suppression of the pathogen and through stimulating the plant defense, by secreting secondary metabolites fengycin and surfactin (Li et al., 2019). In this study, the role of the *N*-acetylglucosaminidase LytD in the biological control of *B. subtilis* GLB191 against grape downy mildew was investigated. We found that LytD affected the direct effect of *B. subtilis* against the pathogen *P. viticola*, the stimulation of the plant defenses, colonization on grape leaves, and, consequently, biocontrol activity against grape downy mildew. These findings not only expand our knowledge on the biological function of autolysins but also provide a new target for improving the biocontrol activity of *Bacillus* and other beneficial microorganisms.

## Materials and methods

### Plant material

The grapevine cultivar *Vitis vinifera* L. cv. Red Globe, susceptible to *P. viticola*, was used in this study. Plant produced from herbaceous cuttings were planted in individual pots at 26 and 18°C (day and night, respectively) with a photoperiod of 16 h of light. The second and third youngest fully expanded leaves were used in the experiments.

### Bacterial strains, plasmids, primers, and growth conditions

The bacterial strains and plasmids used in this study are listed in **Table 1**. *B. subtilis* GLB191 and its derivatives were routinely grown in Luria-Bertani (LB) broth (1% tryptone, 0.5% yeast extract, and 1% NaCl) or on LB agar medium supplemented with 1.5% (w/v) agar at 37°C. For experiments, they were grown in handmade potato dextrose broth (PDB, potato broth supplemented with 1.5% dextrose) medium at 37°C with shaking at 180 r min^−1^ for 48 h. For the biofilm formation assay, *B. subtilis* was cultivated at 30°C in a minimal salts glutamate glycerol (MSgg) medium that was designed to induce biofilm formation. MSgg is composed of 5 mM potassium phosphate (pH 7.0), 100 mM 3-(N-morpholino)propanesulfonic acid (MOPS, pH 7.0), 2 mM MgCl_2_, 50 μM MnCl_2_, 1 μM ZnCl_2_, 50 μM FeCl_3_, 700 μM CaCl_2_, 2 μM thiamine, 0.5% glutamic acid, 50 μg mL^−1^ phenylalanine, 50 μg mL^−1^ threonine, 50 μg mL^−1^ tryptophan, and 0.5% glycerol (Branda et al., 2001). *Escherichia coli* strains were grown in LB broth at 37°C. When appropriate, antibiotics were added at the following concentrations: 100 μg mL^−1^ of ampicillin (Amp) for growth of *E. coli*, 5 μg mL^−1^ of erythromycin (Em), and 10 μg mL^−1^ of tetracycline (Tet) for growth of *B. subtilis*. Primers used for PCR in this report are summarized in **Supplementary Table S1**.

**Table 1:** Strains and plasmids used in this study.

Strains or plasmids	Characteristics ^a^	Sources or references
Strains		
*B. subtilis*		
GLB191	Wild type strain, isolated from grapevine leaves	(Zhang et al., 2017)
*ΔlytD*	*lytD* deletion mutant of GLB191, markerless	This work
*E. coli*		
DH5α	F-φ80 *lac* ZΔM15 Δ(*lacZYA-*arg *F*) *U*169 *endA*1 *recA*1 *hsdR*17(rk-,mk+) *supE*44λ-*thi* -1 *gyrA*96 *relA*1 *phoA*	TakaRa
EC135	EC132 Δ*dam*::*FRT*, genotype of R-M systems: *mcrA* Δ(*mrr*-*hsdRMS*-*mcrBC*) Δ*dcm*::*FRT* Δ*dam*::*FRT*	(Zhang et al., 2012)
Plasmids		
pMAD	Shuttle vector for allele replacement; Amp^R^ (*E. coli*), Em^R^ (*Bacillus*); containing *bgaB* gene encoding a thermostable β-galactosidase	(Arnaud et al., 2004)
pHY300PLK	*Bacillus*-*Escherichia coli* shuttle vector, Amp^R^, Tet^R^	(Ishiwa and Shibahara-Sone, 1986)
pMAD-*lytD*	A fusion of upstream and downstream of *lytD* were cloned into pMAD for allele replacement; Amp^R^ (*E. coli*), Em^R^ (*Bacillus*)	This work
pHY-*lytD*	The *lytD* gene fragment containing its native promoter and ORF were cloned into pHY300PLK plasmid at *Bgl* II/*Sal* I sites; Amp^R^, Tet^R^	This work

### Mutagenesis and complementation of *lytD*

The markerless *lytD* deletion mutant (Δ*lytD*) was constructed by marker exchange mutagenesis using the temperature-sensitive suicide plasmid pMAD as described previously (Li et al., 2019). The flanking regions of *lytD* (GenBank No. PP434428) were amplified from the genomic DNA of GLB191 by polymerase chain reaction (PCR) using the primer pairs *lytD*-up-F/*lytD*-up-R and *lytD*-dn-F/*lytD*-dn-R, respectively. The two DNA fragments were joined together by overlap-extension PCR using the primers *lytD*-up-F and *lytD*-dn-R (Senanayake and Brian, 1995). The resulting fragment was digested and cloned into pMAD, generating pMAD-*lytD* in *E. coli* DH5α. The pMAD-*lytD* plasmid was purified from *E. coli* DH5α and mobilized into *E. coli* EC135 by heat shock and then into GLB191 by electroporation (Zhang et al., 2017). Blue transformants were obtained after incubation at 30°C for 2 days on LB agar plates containing Em and X-Gal (40 μg mL^−1^), followed by incubation in LB broth containing Em at 42°C with shaking at 180 r min^−1^ for 8–10 h for the first allelic exchange. Em-resistant and blue transformants were obtained from LB agar plates supplemented with Em and X-Gal and then incubated in LB broth at 25°C with shaking at 180 r min^−1^ for 24 h for the second allelic exchange. Em-sensitive and white clones were picked and confirmed by PCR amplification and subsequently DNA sequencing to verify the deletion of *lytD* on the chromosome using primers *lytD*-L and *lytD*-D. The primers used are listed in **Supplementary Table S1**.

To complement the Δ*lytD* mutation with the wild-type *lytD* gene, the native promoter and the open reading frame of *lytD* (GenBank No. PP434428) were amplified using primers *lytD*-F/*lytD*-R and then inserted into pHY300PKL, a shuttle plasmid that is replicable in *B. subtilis*, generating the complementary plasmid pHY-*lytD*. pHY-*lytD* was subsequently transformed into Δ*lytD* by electroporation, resulting in complementary strain Δ*lytD* (pHY-*lytD*). pHY300PKL was also transformed into Δ*lytD* and GLB191, respectively, by electroporation to yield strains Δ*lytD*(pHY300) and GLB191(pHY300). Transformants were then selected on 10 μg mL^−1^ Tet-containing LB plates and verified by PCR and DNA sequencing.

### *P. viticola* preparation

The *P. viticola* isolate used for this study was collected from diseased leaves of Muscat Hamburg in the vineyard in Tianjin Academy of Agricultural Sciences and identified by the analysis of the sequence of internal transcribed spacer regions 1 (ITS) (GenBank No. PP413393) as previously described (Rouxel et al., 2013). The *P. viticola* isolate was maintained on Red Globe plants in the glasshouse as previously described (Trouvelot et al., 2008). Sporulation was induced after incubation of plants presenting oily spot symptoms in the dark at >95% relative humidity (RH) overnight. Sporangia were then collected from the lower side of leaves using a brush and suspended in distilled water. The concentration was adjusted to 10^5^ sporangia mL^−1^ using a hemacytometer.

### Biocontrol assay against grape downy mildew

Leaf disc bioassay was carried out to test the biocontrol activity of the bacteria against grape downy mildew. The second and third youngest fully expanded leaves were detached from plants grown in the greenhouse and rinsed with water. Leaf discs of 1 cm diameter were punched from leaves with a cork borer. Discs were soaked in the 48-h culture of GLB191 or its derivatives for 30 min and then plated onto wet paper in Petri dishes with the abaxial side up and air-dried for 2 h. For mock inoculation used as control, the leaf discs were sprayed with PDB medium. One day post-treatment (dpt) with bacterial culture or PDB medium, discs were sprayed with *P. viticola* sporangia suspension at 10^5^ sporangia mL^−1^. Petri dishes were placed overnight in a humid chamber (RH > 95%) and then moved back to the growth chamber at 22°C at a day length of 16 h. Nine discs for each replicate and four replicates for each treatment were used. Three independent biological repeats were conducted.

Disease index (DI) was investigated 5–7 days post-inoculation (dpi). The severity was assessed using the following 0–9 scale as previously described: 0, no lesions; 1, 0%–5% of the leaf disc area infected and sporulating; 3, 6%–25%; 5, 26%–50%; 7, 51%–75%; and 9, more than 76% leaf area infected and sporulating (Zhang et al., 2017).

### Evaluation of a direct effect of bacteria on *P. viticola*

Freshly prepared sporangial suspension of *P. viticola* was used to detect the direct effect of bacteria on the pathogen *in vitro*. *P. viticola* sporangial suspension (500 µL) was incubated with 500 µL of bacterial culture or PDB in 48-well plates in the dark at 18°C until over 50% of sporangia released zoospores for the wild-type strain. Percentage of sporangia released zoospores in the total number of sporangia was visually determined by optical microscopic observation of three different microscopic fields (×400). Three observations for each replicate and three replicates for each treatment were conducted. Counting was repeated on three independent experiments.

Direct effects were also determined on leaves previously treated with bacterial culture as previously described (Li et al., 2019). The lower face of leaves that were treated with bacterial culture or PDB was inoculated with a freshly prepared sporangial suspension (2 × 10^5^ sporangia mL^−1^) 2 h post-treatment (hpt). Leaves were harvested 24 h post-inoculation (hpi) and discs of 0.5 cm diameter were excised from leaves (four discs per leaf) with a cork borer. The discs were subsequently bleached first with pure methanol at least 2 days until completely decolorized and then with chloral hydrate solution (1.0 g L^−1^) for 12–24 h until they become completely transparent. Infection sites were detected after 0.05% aniline blue staining. Three representative fields of each disc and four discs per leaf were observed by ultraviolet (UV) epifluorescence microscopy (Nikon Ti-E, Tokyo, Japan). Three replicates for each treatment and three independent biological repeats were conducted.

### Quantification of callose deposition on grape leaves

The lower face of leaves was treated with cell culture or PDB and harvested 3 dpt. Four discs per leaf (0.5 cm in diameter) were punched, and the tissue was cleared first with pure methanol and subsequently with chloral hydrate solution as described above. Callose deposition was revealed after aniline blue staining as previously described (Li et al., 2019). The number of fluorescent spots (callose deposits) was determined by UV epifluorescence microscopy observations. Three replicates for each treatment and three independent biological repeats were conducted.

### RNA extraction and reverse transcription

The abaxial surface of the second youngest expanded leaves of plants was treated with bacterial culture or PDB and harvested 24 hpt. Four leaves from four plants were pooled and ground in liquid nitrogen. Total plant RNA was isolated using the E.Z.N.A. Plant RNA Kit (OMEGA Bio-Tek, Norcross, GA, USA) according to the manufacturer’s instructions. DNA contaminations were removed using the DNA-free™ kit (Invitrogen, Carlsbad, CA, USA). The concentration of RNA extracts was determined by spectrophotometry. The cDNA was synthesized using the M-MLV Reverse Transcriptase kit (Invitrogen, Carlsbad, CA, USA) through reverse transcription.

### Quantitative RT-PCR

The expression of three genes in grape (*PR2*, *PR3*, and *STS*) known to be upregulated by GLB191 treatment was monitored by qRT-PCR in this study. *EF1γ* encoding the elongation factor 1 gamma was used as the reference gene. The primers are listed in **Supplementary Table S1**. The qRT-PCR procedure was identical to that of the previous report (Li et al., 2019). Briefly, the SYBR Green PCR Master Mix kit (Takara Bio Inc., Shiga, Japan) was used in this study. Relative gene expression was determined with the formula fold induction = 2^−ΔΔCt^, where ΔΔCt = ΔCt (treated sample) − ΔCt (control, i.e., PDB-treated sample) and ΔCt = Ct (target gene) − Ct (reference gene) (Livak and Schmittgen, 2001). Three independent biological repeats were included in each treatment.

### Assay of bacterial colonization on grape leaves

*Bacillus* strains were grown in LB broth at 37°C with shaking at 200 r min^−1^ overnight. Cells were collected after centrifugation at 6,000 × *g* for 20 min at 4°C and washed three times with distilled water. The cells were re-suspended in water and the final bacterial suspension was adjusted to 1×10^7^ cells mL^−1^ for use. Grapevine plants with similar growth status were selected. The bacterial inoculum was sprayed on the second youngest leaves of plants until no droplets dripped off. The control treatment was performed on the same plants by using sterilized water. Three plants were used for each replicate and three replicates were used for each treatment. The persistence of bacteria was monitored at 0, 4, 7, 10, 17, 24, and 31 dpt. Leaves were harvested and washed four times with distilled water. Then, the leaves were then dried with sterile filter paper and subsequently ground with 5 mL of distilled water in a sterile mortar with sterile quartz sand being added to improve the tissue disruption. Samples (100 μL) of plant tissue extracts were diluted by 10-fold serial dilutions in sterile water. One hundred microliters of each diluted suspension was plated on LB agar plates supplemented with Tet. After incubation at 37°C for 12 h, the number of bacterial colony-forming units (CFU g^−1^ fresh leaf weight) was determined. Three independent biological repeats were conducted.

### Scanning electron microscopy

The bacterial colonization on grape leaves was also observed at 31 dpt using scanning electron microscopy (SEM) as described by Sun et al. (2021). Briefly, samples of approximately 1 cm^2^ of leaf blades were fixed with 2.5% dialdehyde in Na-K-phosphate buffer (0.1 M, pH 7. 2) for 2 h at room temperature. The samples were then postfixed in 1% (v/v) OsO_4_ in the PBS (pH 7.2) at room temperature for 2 h and washed three times with PBS (pH 7.2). Subsequently, the samples were dehydrated through an ethanol series (30%, 50%, 70%, 80%, 90%, and 100% for 15 min each). The samples were then critical point dried using a LEICA EM CPD300 (Leica, Mannheim, Germany) and coated with a gold layer using EIKO IB-3 (EIKO, Tokyo, Japan). The bacterial colonization was observed using a HITACHI S-3400N scanning electron microscope (Hitachi, Tokyo, Japan).

### Biofilm formation assay

The bacterial cell culture was grown in 5 mL of LB liquid medium supplemented with Tet overnight and then transferred to 5 mL of fresh LB medium to grow to mid-log phase (OD_600_ 0.8–1.0). Bacterial cell culture (4 µL) was added to 4 mL of MSgg medium in a 12-well microtiter plate and incubated at 30°C for 48–72 h. Images were taken using a Nikon BM-7 digital camera (Nikon Corporation, Tokyo, Japan). Four replicates were set for each treatment. Three independent biological repeats were conducted.

### Statistical analysis

Analysis of variance (ANOVA) was performed with the statistical program SPSS software 21.0 using the Duncan test or Student’s *t*-test to assess significant differences between treatments (*p* < 0.05).

## Results

### *N*-acetylglucosaminidase LytD plays an important role in the protection of grapevine by GLB191 against downy mildew

**Figure 1:**
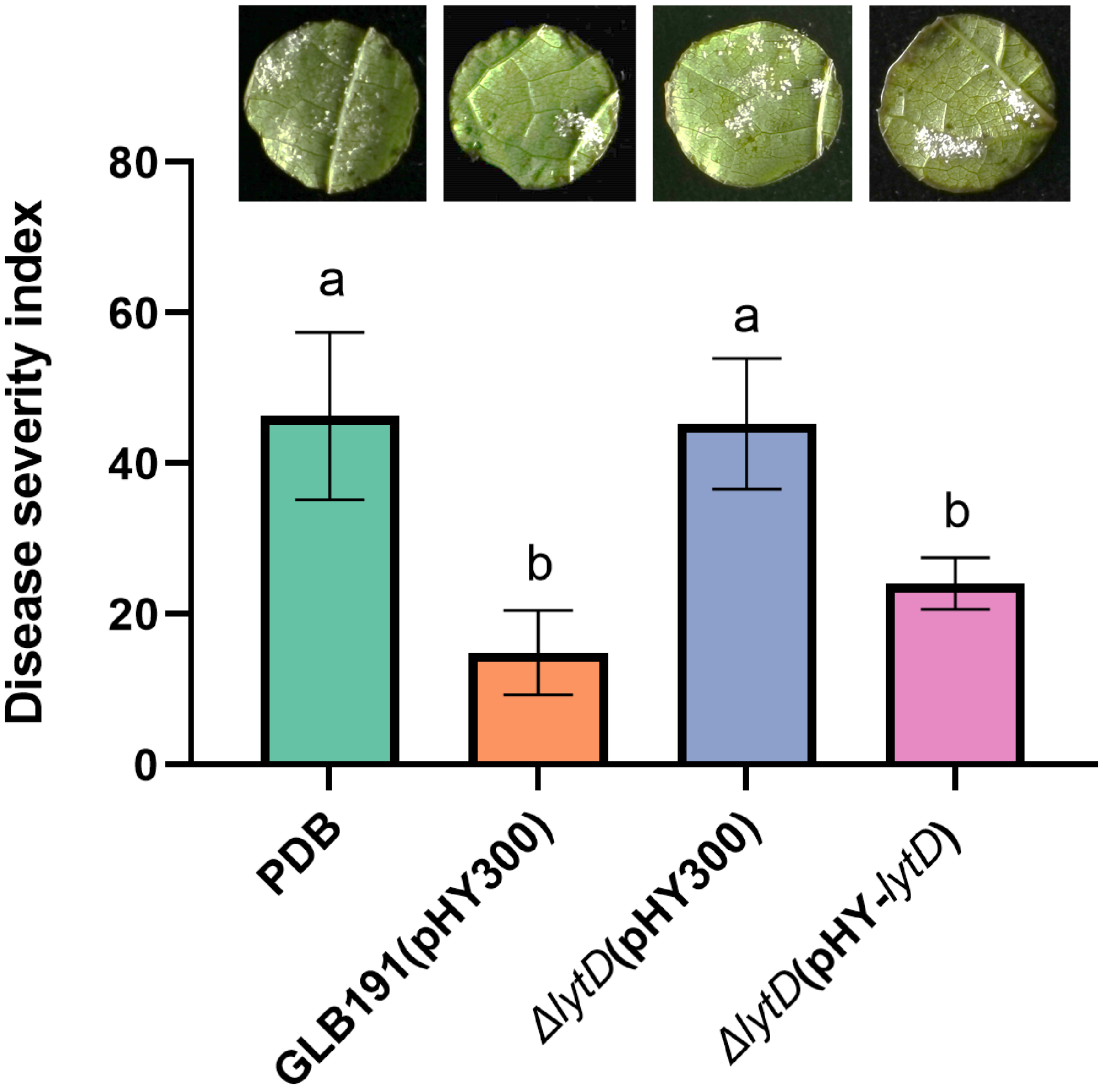
Effect of *lytD* on the protection of grapevine leaves against grape downy mildew induced by *B*. *subtilis* GLB191. Disease severity was assessed with the grape downy mildew on susceptible cultivar cv. Red Globe treated with PDB medium (PDB) or the 48-h culture of the wild-type *B*. *subtilis* GLB191(pHY300) [GLB191(pHY300)], Δ*lytD*(pHY300), and Δ*lytD*(pHY-*lytD*). Values are the means ± standard deviations (SDs) obtained from 36 discs of four replicates. The data are representative of three independent experiments. Different letters on the column indicate significant differences according to the Duncan test (*p* < 0.05).

To test whether *N*-acetylglucosaminidase LytD is involved in the biocontrol efficacy of *B. subtilis* GLB191 (hereafter called GLB191) against grape downy mildew, *lytD* deletion mutant (Δ*lytD*) was constructed and its biocontrol activity was compared with the wild-type strain. Foliar treatment of an *in vitro* culture of the wild-type GLB191 significantly suppressed the DI of downy mildew on grapevine compared to the medium-only treatment. However, the biocontrol activity of GLB191 was significantly compromised upon deletion of *lytD* (**Figure 1**), suggesting that LytD is important for the biocontrol activity of *B. subtilis* GLB191 against *P. viticola*. The reduced biocontrol activity of Δ*lytD* can be complemented by pHY-*lytD* (**Figure 1**), which confirms our conclusion. These results suggest that *N*-acetylglucosaminidase LytD plays an important role in the biocontrol of GLB191 against *P. viticola*.

### LytD of *B. subtilis* impacts its direct effect on *P. viticola*

**Figure 2:**
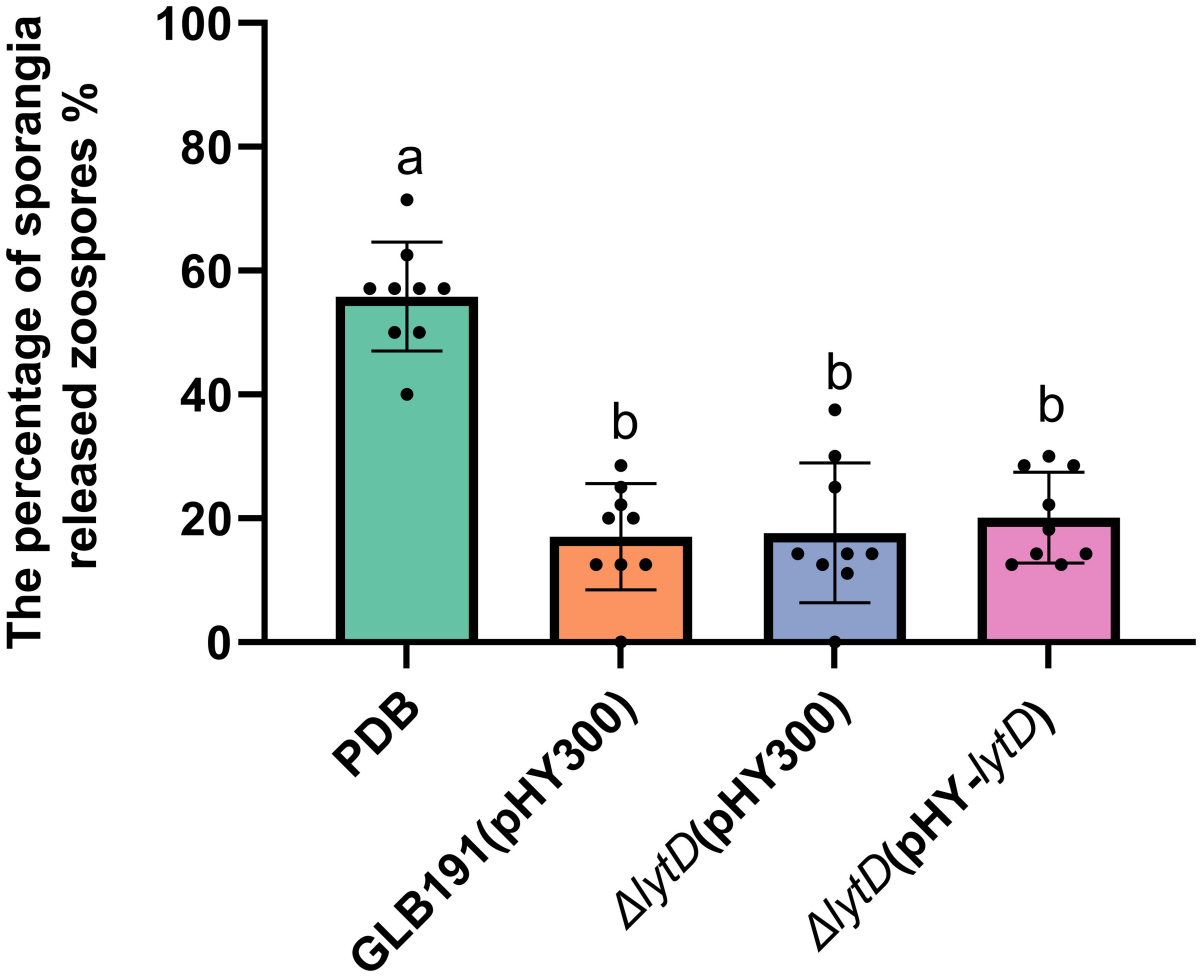
Direct effect of *B*. *subtilis* GLB191 and its derivatives on *P. viticola* sporangia releasing zoospores *in vitro*. *B*. *subtilis* GLB191(pHY300) [GLB191(pHY300)], Δ*lytD*(pHY300), and Δ*lytD*(pHY-*lytD*) were cultured in PDB broth for 48 h at 37°C. *P. viticola* sporangial suspension (500 µL) was incubated with 500 µL of bacterial culture or PDB for 1–3 (h) The percentage of sporangia released zoospores was determined by optical microscopic observations. Data are means ± SD from nine observations of three replicates. Different letters on the column indicate significant differences according to the Duncan test (*p* < 0.05).

The biocontrol activity of GLB191 against downy mildew disease results from both direct suppression of the pathogen *P. viticola* and stimulation of the plant defense (Li et al., 2019). In order to further unravel the mechanisms of LytD in biocontrol of GLB191, we first examined the impact of *lytD* deletion on *P. viticola* growth under *in vitro* conditions. Percentage of sporangia released zoospores of *P. viticola* was determined in the presence and absence of the GLB191 strains. Wild-type GLB191 treatment significantly reduced the percentage of sporangia released zoospores compared to the medium-only treatment (**Figure 2**), indicating that GLB191 inhibits the viability of sporangia. Comparable percentage of sporangia released zoospores was observed in Δ*lytD* and Δ*lytD*(pHY-*lytD*) as compared to the wild-type GLB191. These results suggest that LytD did not impact the effect of *B. subtilis* on the sporangia viability under *in vitro* conditions.

**Figure 3:**
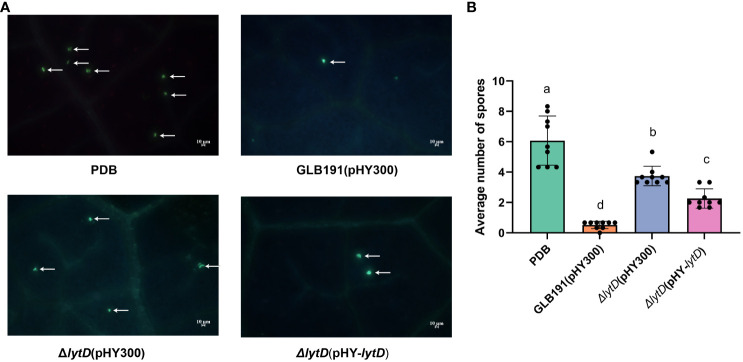
Direct effect of *B*. *subtilis* GLB191 and its derivatives on *P. viticola* zoospores on grapevine leaves. *B*. *subtilis* GLB191(pHY300) [GLB191(pHY300)], Δ*lytD*(pHY300), and Δ*lytD*(pHY-*lytD*) were cultured in PDB medium for 48 h at 37°C. Grapevine (*V. vinifera* cv. Red Globe) leaves were treated with PDB medium (PDB) or the culture of GLB191(pHY300), Δ*lytD*(pHY300), and Δ*lytD*(pHY-*lytD*), and inoculated with *P. viticola* sporangia 2 h post-treatment. Leaf discs were then punched out from leaves 24 h post-inoculation, and the number of infection sites (i.e., stomata with encysted zoospores of *P. viticola*) was determined by UV epifluorescence observations after aniline blue staining of the pathogen. **(A)** Representative fields of fluorescence microscopy observations. Arrows indicate the infection sites (encysted zoospores). Scale bars represent 10 μm. **(B)** Values are the mean number of infection sites (means ± SD) for 9 discs from three replicates. Different letters on the column indicate significant different at *p* < 0.05 according to the Duncan test. The data are representative of three independent experiments.

The anti-oomycete effect of GLB191 and Δ*lytD* was further compared under *in vivo* conditions. The number of infection sites represented by the number of stomata with encysted zoospores of *P. viticola* was determined at 24 hpi by UV epifluorescence observations after aniline blue staining of the pathogen. The highest number of infection sites (6.0 ± 1.6 per observation field) was observed for the PDB media-only treatment (**Figure 3**). Consistent with previous results, almost no infection sites (0.5± 0.2 per observation field) could be observed on leaves treated with wild-type GLB191 (**Figure 3**), suggesting that GLB191 has a significant direct anti-oomycete effect on zoospores. Interestingly, deletion of *lytD* resulted in a significantly higher number of infection sites (3.7 ± 0.6 per observation field) compared to the wild type. Complementing the Δ*lytD* strain with an *in trans lytD* gene partially restored this phenotype to a comparable level of the wild-type GLB191 (**Figure 3**). The results above indicate that *N*-acetylglucosaminidase LytD of *B. subtilis* GLB191 is required for its anti-oomycete effect against *P. viticola* zoospores infection although it does not affect its antagonism of sporangia viability.

### LytD contributes to the induction of defense responses by *B. subtilis*

**Figure 4:**
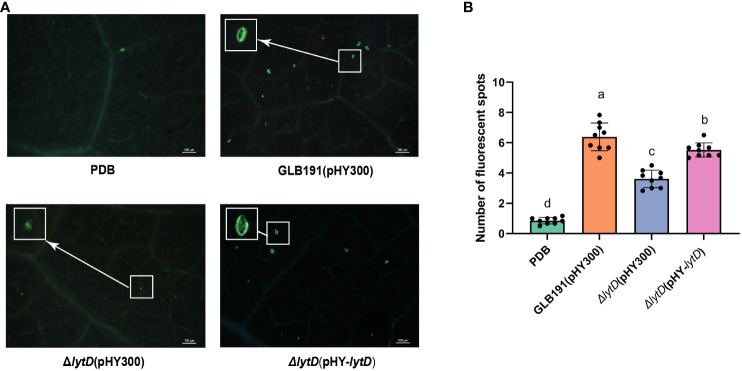
Callose deposition induced by *B*. *subtilis* GLB191 and its derivatives on grapevine leaves. Grapevine (*V. vinifera* cv. Red Globe) leaves were treated with PDB medium (PDB) or the culture of the wild-type *B*. *subtilis* GLB191(pHY300) [GLB191(pHY300)], Δ*lytD*(pHY300), and Δ*lytD*(pHY-*lytD*). Callose production (fluorescent spots) was observed 3 days post-treatment by epifluorescence observations after aniline blue staining. **(A)** Representative fields observed using a fluorescence microscope. Scale bars represent 100 μm. **(B)** Values are the mean number of fluorescent spots (means ± SD) for 9 discs from three replicates. Different letters on the column indicate significant difference at *p* < 0.05 according to the Duncan test. The data are representative of three independent experiments.

In order to further determine whether *lytD* is involved in inducing defense responses of grapevine leaves, callose production and defense gene expression measured by qRT-PCR were chosen as two representative phenotypical markers of plant defense to be evaluated upon treatment of wild-type GLB191 and Δ*lytD* (Li et al., 2019). The *in vitro* culture of *Bacillus* strains was sprayed on grape leaves and callose production was monitored at 3 dpi by UV epifluorescence after aniline blue staining. As anticipated, the number of fluorescent spots was significantly increased after treatment by GLB191 (6.4 ± 0.9 per observation field) as compared to the media-only treatment (0.9 ± 0.2 per observation field) (**Figure 4**), indicating a strong induction of callose production by GLB191. In contrast, disruption of *lytD* resulted in a marked decrease in the number of spots (3.6 ± 0.6 per observation field) (**Figure 4**). This phenotype can be complemented by Δ*lytD*(pHY-*lytD*) (5.5 ± 0.5 per observation field) (**Figure 4**), thus confirming the important role of *lytD* in inducing callose deposition in grapevine leaves.

**Figure 5:**
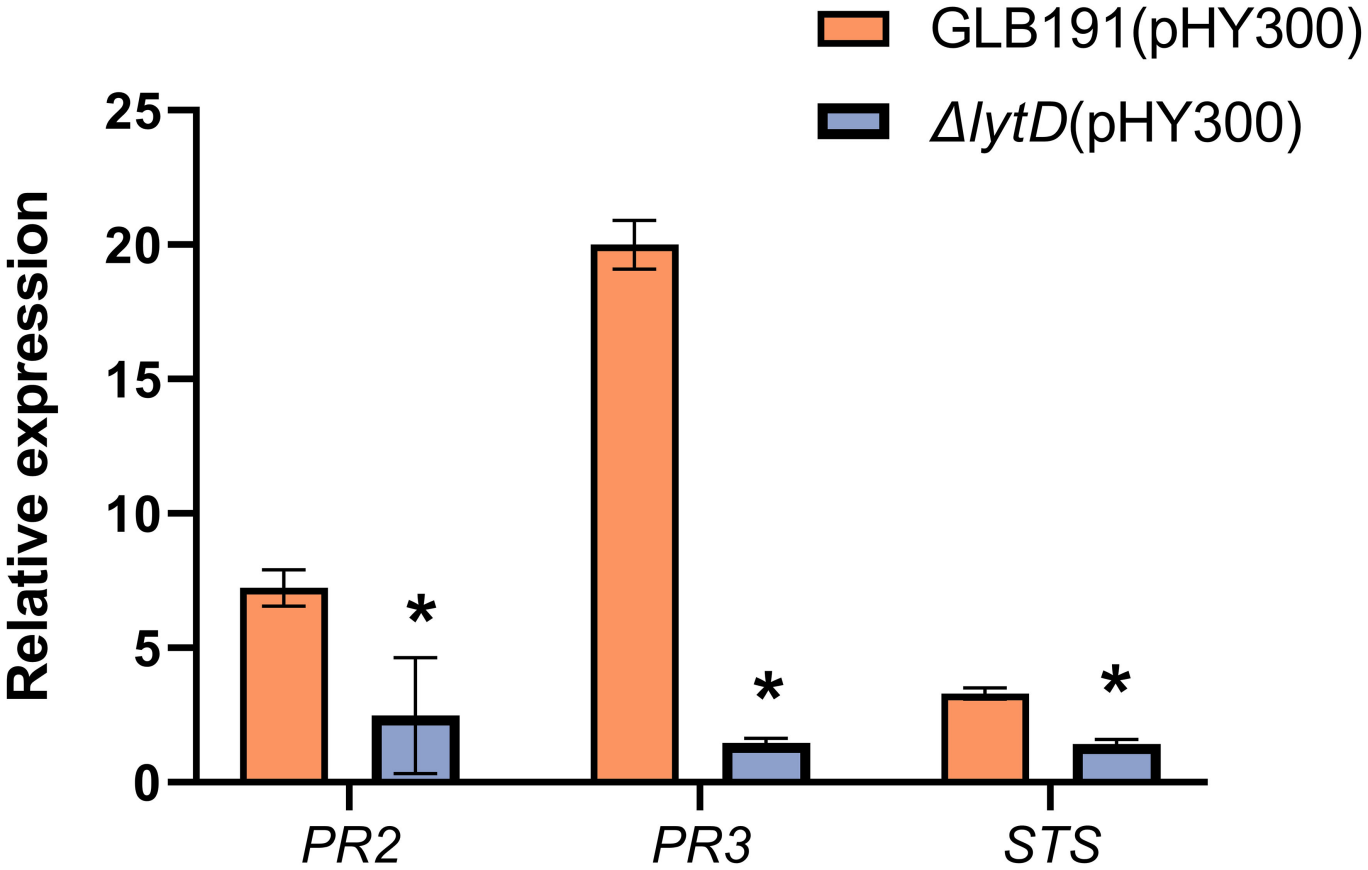
Defense-related gene expression in grapevine leaves treated with *B*. *subtilis* GLB191 and its derivatives. Grapevine (*V. vinifera* cv. Red Globe) leaves were treated with PDB medium (PDB) or the culture of the wild-type *B*. *subtilis* GLB191(pHY300) [GLB191(pHY300)] and Δ*lytD*(pHY300) and harvested 24 h post-treatment. The relative transcript accumulation of defense genes *PR2* (encoding the pathogenesis-related protein 2 β-1,3-glucanase), *PR3* (encoding the pathogenesis-related protein 3 chitinase 4c), and *STS* (encoding stilbene synthase) was determined by qRT-PCR. Values are the fold increase in transcript level compared to the PDB-treated leaves. Data represent mean ± SD of triplicate measurements from a representative experiment. Asterisks above the column indicate significant difference in transcript level in leaves treated with Δ*lytD*(pHY300) compared to that of GLB191(pHY300)-treated ones at *p* < 0.05 according to Student’s *t*-test.

Next, the expression of three plant defense-related genes, *PR2* (encoding the PR protein 2 β-1,3-glucanase), *PR3* (encoding the PR protein 3 chitinase 4c), and *STS* (encoding stilbene synthase, downstream of PAL and responsible for the synthesis of resveratrol, the main phytoalexin produced by grapevine in response to biotic or abiotic stresses), previously known to be induced by GLB191 (Li et al., 2019) was evaluated upon deletion of *lytD*. As expected, *PR2*, *STS*, and *PR3* were highly upregulated upon GLB191 treatment by 8.5-, 20.2-, and 3.8-fold, respectively (**Figure 5**). However, the transcript accumulation of these defense genes was only 2.0-, 1.5-, and 1.4-fold relative to PDB in response to Δ*lytD* treatment (**Figure 3**), suggesting that LytD contributes to the induction of defense gene expression after GLB191 treatment. Taken together, the results above indicate that LytD is involved in the induction of plant defense response by *B. subtilis* GLB191.

### Disruption of *lytD* does not affect bacterial growth under *in vitro* conditions but reduced bacterial colonization on grape leaves

**Figure 6:**
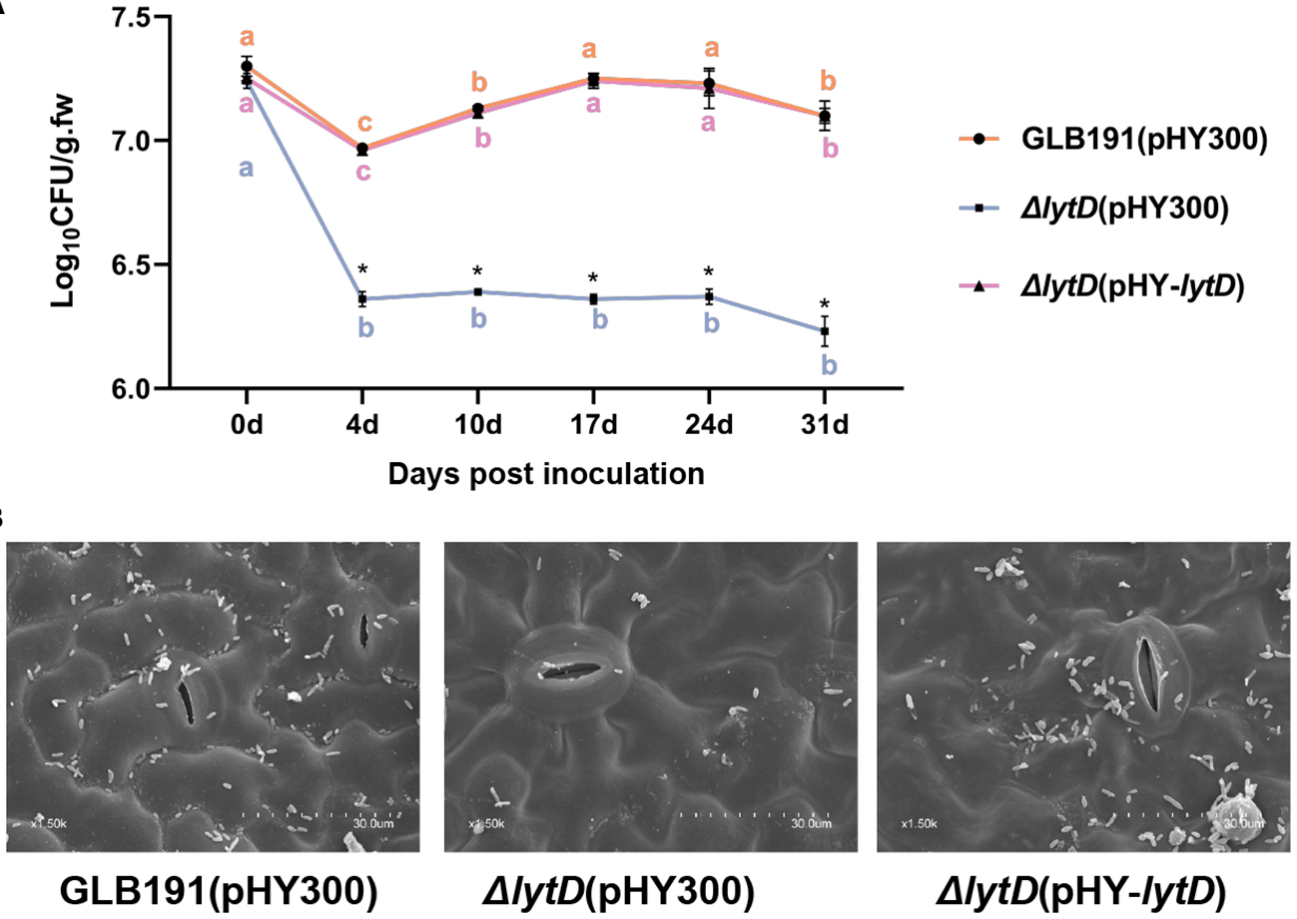
Colonization of *B*. *subtilis* GLB191 and its derivatives on grapevine leaves. Grapevine (*V. vinifera* cv. Red Globe) leaves were treated with the cells of the wild-type *B*. *subtilis* GLB191(pHY300) [GLB191(pHY300)], Δ*lytD*(pHY300), and Δ*lytD*(pHY-*lytD*). **(A)** Bacterial population was isolated from grapevine leaves at 0, 4, 7, 10, 17, 24, and 31 days post-treatment and determined through plate CFU counts. Values correspond to the mean bacterial population (CFU g^−1^ fresh weight) (mean ± SD) from three grapevine leaves. The data are representative of three independent experiments. Different letters in the same color on the curve indicate significant differences between different time points for the same strain according to the Duncan test (*p* < 0.05). Asterisks above the curve indicate significant difference in bacterial population at the same time point between different strains according to Student’s *t*-test (*p* < 0.05). **(B)** Bacterial colonization on grapevine leaves observed at 31 days post-treatment through scanning electron microscopy.

Sufficient colonization of biocontrol microorganism on plants is as an important first step required for the subsequent biocontrol activities (Sachdev and Singh, 2018). We next determined whether LytD affects the growth of *B. subtilis* GLB191 under *in vitro* conditions and, subsequently, its colonization on grape leaves. Cultured in LB medium, the growth of GLB191(pHY300), Δ*lytD*(pHY300), and Δ*lytD*(pHY-*lytD*) strains was similar to each other within the test period of 19 h (**Supplementary Figure S1**). Interestingly, the population of *B. subtilis* GLB191 determined by plating on LB medium supplemented with the antibiotic Tet revealed a reduction in *B. subtilis* colonization on grape leaves upon deletion of Δ*lytD*. Compared to wild-type *B. subtilis* GLB191 that showed an initial decrease (4 dpi), followed by a significant increase (17 dpi) and then stability (24 dpi) (**Figure 6A**), Δ*lytD* showed a dramatic reduction in its population except on 0 dpi. The reduced plant colonization phenotype could be restored by the complementary strain Δ*lytD*(pHY-*lytD*) (**Figure 6A**). No bacteria were recovered on the LB containing tetracycline from the uninoculated plants throughout the experiments (data not shown). These results suggest that the LytD is important for grape leaf colonization although this is not due to the growth impediment.

The reduced plant colonization was confirmed by SEM. Heavy colonization of GLB101 was observed at 31 dpi on the surface of the GLB191-treated leaves. In contrast, markedly less bacteria were observed on the surface of the Δ*lytD*-treated leaves. When introducing a complementary plasmid pHY-*lytD* into Δ*lytD*, the bacteria amount was almost restored to the wild-type level (**Figure 6B**). These results confirm that LytD is important for *B. subtilis* GLB191 colonization on grape leaves.

### Inactivation of LytD decreased the biofilm formation of *B. subtilis*

**Figure 7:**
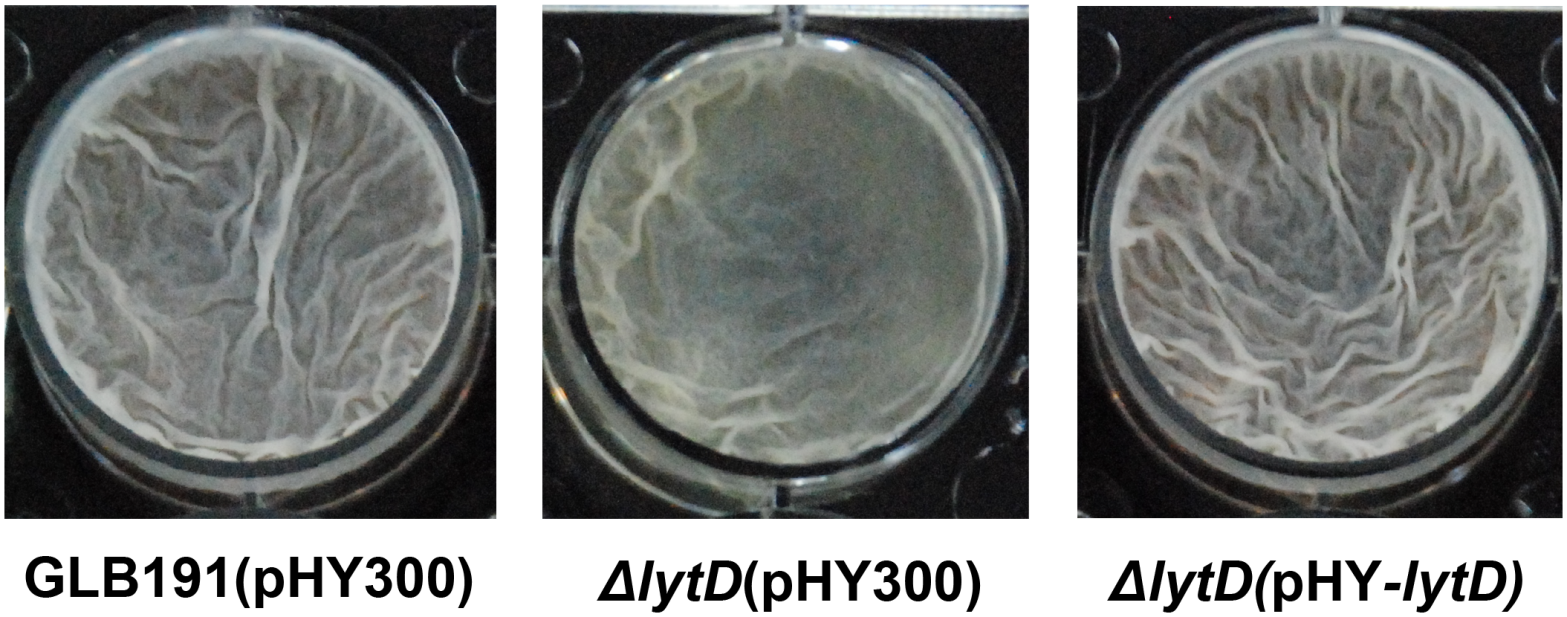
Biofilm formation of *B*. *subtilis* GLB191 and its derivatives. The wild-type *B*. *subtilis* GLB191(pHY300) [GLB191(pHY300)], Δ*lytD*(pHY300), and Δ*lytD*(pHY-*lytD*) were grown in LB at 37°C overnight and then transferred to fresh LB to grow to mid-log phase (OD_600_ 0.8–1.0). Cultures (4 μL) were added into 4 mL of MSgg medium in a 12-well microtiter plate and incubated at 30°C. Images were taken using a Nikon BM-7 digital camera after 48–72 h.

It is widely recognized that biofilm formation is critical for the colonization of bacteria on plant surfaces, especially for *Bacillus* species (Chen et al., 2013; Arnaouteli et al., 2021; Gong et al., 2022). To determine whether the impaired plant colonization of Δ*lytD* is due to a reduced biofilm formation, we measured the pellicle biofilm formation of the wild type and Δ*lytD* of GLB191 in MSgg medium. Compared to wild-type GLB191 that formed a strong biofilm in MSgg medium, the disruption of *lytD* led to reduced biofilm formation. The biofilm formation of the complementary strain Δ*lytD*(pHY-*lytD*) was restored to the wild-type level (**Figure 7**). These results suggest that LytD is involved in *B. subtilis* GLB191 biofilm formation.

**Figure 8:**
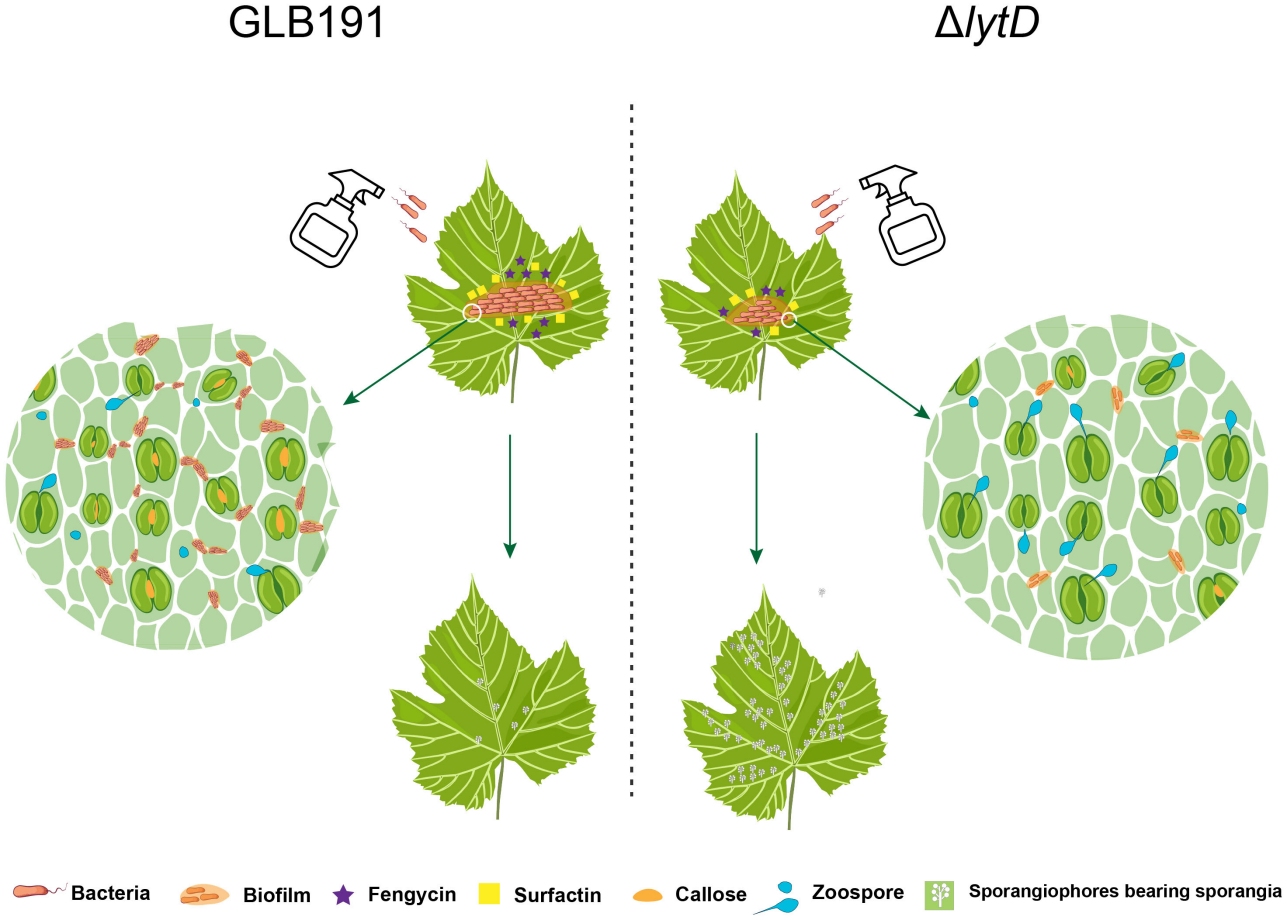
Model of LytD regulation on the biocontrol efficacy of *B*. *subtilis* GLB191 against grape downy mildew. Upon landing on a grapevine leaf, vegetative cells of *B*. *subtilis* GLB191 (GLB191) produce the antimicrobial lipopeptide surfactin (Srf) to antagonize the pathogen *P. viticola* and to activate biofilm formation and further colonize grapevine leaf. Besides surfactin, it also produces fengycin (Fen). Both surfactin and fengycin contribute to inhibit the infection (germination) of *P. viticola* and the induction of defense resistance, which was associated with high callose deposition. Inactivation of *lytD* (Δ*lytD*) resulted in decreased biofilm formation and the consequent colonization on grape leaves, which determine its efficiency to direct effect on the pathogen and induction of defense responses, thus reducing biocontrol activity against grape downy mildew.

In summary, the roles of LytD in biological control of *B. subtilis* are depicted in **Figure 8**. LytD positively impacts bacterial biofilm formation and thereby colonization on grape leaves, which determine its efficiency to stimulate the plant defenses and direct effect on the pathogen, and finally impact its biocontrol activity.

## Discussion

*B. subtilis* is one of the most widely studied biocontrol agents (BCAs). Many of the factors, genes, and conditions needed by this organism to protect plants are well studied. However, many aspects of the regulation of the protection are not fully elucidated. In this study, the autolysin *N*-acetylglucosaminidase LytD, an endogenous CWDE of *B. subtilis* acting on the peptidoglycan layer of its own cell wall, was found to play an important role in the biocontrol activity of *B. subtilis* against grape downy mildew. Evidence also showed that *N*-acetylglucosaminidase LytD of *B. subtilis* regulates its biocontrol activity through biofilm formation and the consequent colonization on grape leaves, which determine its direct effect on the pathogen and induction of defense responses. This is the first study to report on the role of an endogenous CWDE in biological control.

Competent colonization is the most important prerequisite towards efficient biocontrol (Sachdev and Singh, 2018). An efficient BCA must be able to establish itself and survive on plant. Therefore, the first step towards an efficient biocontrol is to demonstrate the successful colonization on the plant (Maurer et al., 2013). There is ongoing research to identify genes or factors needed for colonization. Among these studies, biofilm is at the top, which plays essential roles in the colonization of the BCA after inoculation (Fessia et al., 2022). GLB191 has a strong protective activity against grape downy mildew in both leaf disk assays and the field (Zhang et al., 2017). As expected, it also showed strong biofilm formation and exceptionally high and stable cell densities on grapevine leaves (**Figures 6**, **7**), indicating competent colonization on grapevine leaves, which makes it a promising candidate for BCA.

The cell wall is the first line of defense against pathogen attacks. BCAs produce an array of CWDEs to degrade the cell wall of phytopathogenic fungi. For example, *B. subtilis* produces chitinases and glucanases, which are important in the biological control of phytopathogenic fungi (Manjula and Podile, 2005; Shrestha et al., 2015). Additionally, CWDEs can act as elicitors of host defense reaction (Kikot et al., 2009; Brunner et al., 2013; Kubicek et al., 2014; Tayi et al., 2016; Bacete et al., 2018; Giovannoni et al., 2020). Moreover, CWDEs also contribute to the colonization of bacteria on plants. For example, endo-β-1,4-glucanase of *B. amyloliquefaciens* is needed for its optimal endophytic colonization (Fan et al., 2016). In this study, we found that autolysin *N*-acetylglucosaminidase LytD of *B. subtilis* plays an important role in the suppression of the pathogen *P. viticola*, stimulation of the plant defense, biofilm formation and colonization, and the biocontrol activity against grape downy mildew (**Figures 1**–**7**). These results are in accordance with previous reports, indicating that endogenous CWDEs also play important roles in multiple aspects besides cell development. These findings expand our knowledge on the biological function of autolysins and provide a new target to promote the biocontrol activity of *B. subtilis*.

Even though the direct effect on the pathogen and the stimulation of the defense, which are the main mechanisms of *B. subtilis* GLB191 to protect grapevine against downy mildew (Li et al., 2019), reduced the suppression of the pathogen *P. viticola* and the stimulation of the plant defense by the inactivation of LytD, they are probably not the direct reasons for its reduced biocontrol efficacy since colonization is a prerequisite towards efficient biocontrol among all mechanisms. Given that the bacterial growth rate under *in vitro* conditions and the percentage of sporangia released zoospores *in vitro* were not significantly affected by disruption of *lytD* (**Figure 2**; **Supplementary Figure S1**), we speculate that the reduction of biofilm and, therefore, colonization are the direct reasons for its reduced biocontrol efficacy in Δ*lytD*. Therefore, we propose that *N*-acetylglucosaminidase LytD of *B. subtilis* regulates its biocontrol efficacy against grape downy mildew through colonization. However, the effect of LytD on the production of surfactin and fengycin, which are the main active compounds for its biocontrol efficacy against grape downy mildew, still needs to be determined. In addition, it is worth determining whether *lytD* was induced when *B. subtilis* interacts with the grapevine leaves.

The genome of *B. subtilis* harbors at least 35 autolysin genes. Although autolysins associated with *B. subtilis* have been known for many years, the study of their physiological roles has been hampered by their great number and functional redundancy (Smith et al., 2000). Therefore, analysis of multiply inactivated mutants provides the possibility to define the roles played by individual autolysins in a number of important cellular processes. LytC and LytD are two major autolysins expressed during the vegetative phase of growth in *B. subtilis* 168. Both enzymes are dispensable for growth but have mutually compensatory roles in cell wall turnover, motility, and cell separation (Rashid et al., 1995; Ren et al., 2022). Inconsistent with *B. subtilis* 168, single deletion of *lytD* of *B. subtilis* GLB191 showed significant phenotype, probably due to the different strain. Therefore, it will be important to identify and analyze the total complement of autolysins to determine their individual and combined roles in *B. subtilis* GLB191. It will provide a better understanding of the complex roles in biological control played by the autolysins of *B. subtilis* GLB191.

## Data availability statement

The raw data supporting the conclusions of this article will be made available by the authors, without undue reservation.

## Author contributions

LTW: Formal analysis, Investigation, Methodology, Software, Writing – original draft. JQH: Methodology, Resources, Supervision, Writing – review & editing. SC: Methodology, Resources, Review & editing. XS: Methodology, Software, Writing – editing. XZ: Resources, Writing – review & editing. LJW: Resources, Review – editing. WZ: Writing – review & editing. ZSW: Methodology, Review – editing. QCZ: Methodology, Review – editing. QW: Resources, Supervision, Review – editing. LY: Conceptualization, Methodology, Supervision, Writing – review & editing.
