# Supplementary material for: Endogenous cell wall degrading enzyme LytD is important for the biocontrol activity of Bacillus subtilis
Source: Front Plant Sci. 2024 Apr 10;15:1381018. doi: 10.3389/fpls.2024.1381018 (PMC11039861; doi:10.3389/fpls.2024.1381018)
Supplement: Supplementary file 1 [file DataSheet_1.docx]

Supplementary Material

# Supplementary Figures and Tables

## Supplementary Figures
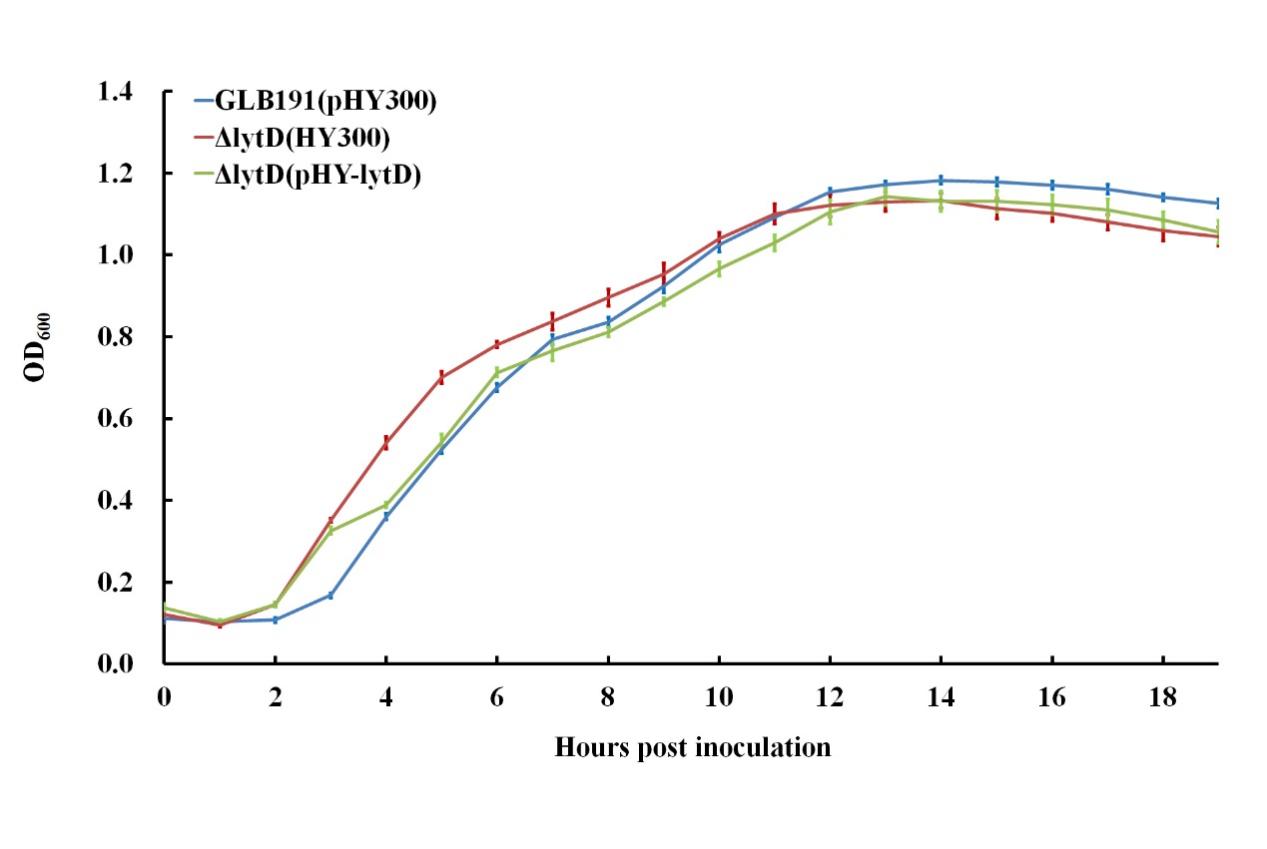


**Supplementary Figure S1. The growth rate of the wild type *B. subtilis* GLB191(pHY300) [GLB191(pHY300)], Δ*lytD*(pHY300) and Δ*lytD*(pHY-*lytD*).**

## Supplementary Tables

# Supplementary Table S1 Primes used in the study.

| **Primer** | **Targent gene** | **Sequence (5’-3’) (restriction sites underlined)** | **Amplicon** | **Reference** |
| --- | --- | --- | --- | --- |
| *lytD*-up-F | *lytD* | GAAGATCTCGGAACCGCTTTACGAGATAGATTT | *lytD* deletion | This study |
| *lytD*-up-R |  | AAACACCTTTTGCAGCCGCAGCTTCATTTATAGGTGAATCACT |  | This study |
| *lytD*-dn-F |  | AGTGATTCACCTATAAATGAAGCTGCGGCTGCAAAAGGTGTTT |  | This study |
| *lytD*-dn-R |  | CGACGCGTAACTGATGTCGATGGCTTAT |  | This study |
| *lytD*-L | *lytD* | GTTCTACGATTTCAGAGGAAGGTTCTTC | *lytD* deletion comfirmation | This study |
| *lytD*-D |  | TCAATTGATACTTCAAATGTGCCTGGAGCC |  | This study |
| *lytD*-F | *lytD* | GAAGATCTTCACGGGTATATTGATGAATCGACC | *lytD* complementation | This study |
| *lytD*-R |  | ACGCGTCGACGTCGGCCATAGCGGCCGCGGAAAACGTCGGCGTTTCCACAA |  | This study |
| EF1ɣ F | elongation factor 1 chain gamma | GAAGGTTGACCTCTGGGATG | qRT-PCR | ([Gamm et al., 2011](#_ENREF_3); [Li et al., 2019](#_ENREF_4)) |
| EF1ɣ R |  | AGAGCCTCTCCCTCAAAAGG |  |  |
| PR2 F | class I beta-1,3-glucanase | ATGCTGGGTGTCCCAAACTCG | qRT-PCR | ([Aziz et al., 2003](#_ENREF_1); [Dubreuil-Maurizi et al., 2010](#_ENREF_2); [Li et al., 2019](#_ENREF_4)) |
| PR2 R |  | CAGAACAAACTGCGCAAACCGT |  |  |
| PR3 F | class IV chitinase (CHI4C) | GCAACCGATGTTGACATATCA | qRT-PCR | ([Aziz et al., 2003](#_ENREF_1); [Li et al., 2019](#_ENREF_4)) |
| PR3 R |  | CGTCGCCCTAGCAAGTGAG |  |  |
| STS F | stilbene synthase | AGGAAGCAGCATTGAAGGCTC | qRT-PCR | ([Trouvelot et al., 2008](#_ENREF_5); [Li et al., 2019](#_ENREF_4)) |
| STS R |  | TGCACCAGGCATTTCTACACC |  |  |

Reference:

Aziz, A., Poinssot, B., Daire, X., Adrian, M., Bézier, A., Lambert, B., et al. (2003). Laminarin elicits defense responses in grapevine and induces protection against *Botrytis cinerea* and *Plasmopara viticola*. *Molecular Plant-Microbe Interactions* 16(12)**,** 1118-1128. doi: Doi 10.1094/Mpmi.2003.16.12.1118.

Dubreuil-Maurizi, C., Trouvelot, S., Frettinger, P., Pugin, A., Wendehenne, D., and Poinssot, B. (2010). β-Aminobutyric acid primes an NADPH oxidase-dependent reactive oxygen species production during grapevine-triggered immunity. *Molecular Plant-Microbe Interactions* 23(8)**,** 1012-1021. doi: 10.1094/Mpmi-23-8-1012.

Gamm, M., Héloir, M.C., Kelloniemi, J., Poinssot, B., Wendehenne, D., and Adrian, M. (2011). Identification of reference genes suitable for qRT-PCR in grapevine and application for the study of the expression of genes involved in pterostilbene synthesis. *Molecular Genetics and Genomics* 285(4)**,** 273-285. doi: 10.1007/s00438-011-0607-2.

Li, Y., Heloir, M.C., Zhang, X., Geissler, M., Trouvelot, S., Jacquens, L., et al. (2019). Surfactin and fengycin contribute to the protection of a *Bacillus subtilis* strain against grape downy mildew by both direct effect and defence stimulation. *Molecular Plant Pathology* 20(8)**,** 1037-1050.

Trouvelot, S., Varnier, A.-L., Allègre, M., Mercier, L., Baillieul, F., Arnould, C., et al. (2008). A β-1, 3 glucan sulfate induces resistance in grapevine against *Plasmopara viticola* through priming of defense responses, including HR-like cell death. *Molecular Plant-Microbe Interactions* 21(2)**,** 232-243.
